# Hsp90 interaction networks in fungi—tools and techniques

**DOI:** 10.1093/femsyr/foab054

**Published:** 2021-10-28

**Authors:** Julia L Crunden, Stephanie Diezmann

**Affiliations:** School of Cellular and Molecular Medicine, University of Bristol, University Walk, Bristol BS8 1TD, UK; School of Cellular and Molecular Medicine, University of Bristol, University Walk, Bristol BS8 1TD, UK

**Keywords:** protein–protein interactions, proteomics, chemogenomics, Hsp90, synthetic lethality, mutant libraries, *Candida*, *Cryptococcus neoformans*, *Aspergillus fumigatus*

## Abstract

Heat-shock protein 90 (Hsp90) is a central regulator of cellular proteostasis. It stabilizes numerous proteins that are involved in fundamental processes of life, including cell growth, cell-cycle progression and the environmental response. In addition to stabilizing proteins, Hsp90 governs gene expression and controls the release of cryptic genetic variation. Given its central role in evolution and development, it is important to identify proteins and genes that interact with Hsp90. This requires sophisticated genetic and biochemical tools, including extensive mutant collections, suitable epitope tags, proteomics approaches and Hsp90-specific pharmacological inhibitors for chemogenomic screens. These usually only exist in model organisms, such as the yeast *Saccharomyces cerevisiae*. Yet, the importance of other fungal species, such as *Candida albicans* and *Cryptococcus neoformans*, as serious human pathogens accelerated the development of genetic tools to study their virulence and stress response pathways. These tools can also be exploited to map Hsp90 interaction networks. Here, we review tools and techniques for Hsp90 network mapping available in different fungi and provide a summary of existing mapping efforts. Mapping Hsp90 networks in fungal species spanning >500 million years of evolution provides a unique vantage point, allowing tracking of the evolutionary history of eukaryotic Hsp90 networks.

## Teaser

Hsp90 networks in fungi provide a unique opportunity for the study of network evolution.

## INTRODUCTION

Proteins facilitate diverse cellular processes, such as metabolism, the electron transport chain and movement of organelles. It is, thus, critical that proteins remain folded and stable, yet they are inherently unstable. Their folding is maintained by the equivalent energy of approximately three hydrogen bonds, meaning they exist on a thermodynamic knife-edge. As such they are vulnerable to denaturation or aggregation, especially in the crowded cellular environment (Ellis 2001) or when cells are experiencing environmental stress. In response to these challenges, chaperone proteins evolved to assist with protein folding and stabilization, thereby maintaining proteostasis and ultimately ensuring survival of the organism.

### Hsp90 is an essential chaperone and evolutionary capacitor

The highly conserved and ubiquitous molecular chaperone Heat-shock protein 90 (Hsp90) was first described in the 1970s as a protein of approximately 90 kDa that was significantly upregulated during heat stress in *Drosophila* cell culture and salivary gland tissue (Moran *et al*. 1978). Subsequently, orthologs have been identified in eubacteria and all eukaryotes but not archaebacteria (Stechmann and Cavalier-Smith 2004). Hsp90 protein sequences are 60% identical between human and the model eukaryote *Saccharomyces cerevisiae*. Mammalian Hsp90 can complement the otherwise lethal phenotype of Hsp90 depletion in *S. cerevisiae* (Minami *et al*. 1994; Nathan, Harju Vos and Lindquist 1999). Hsp90 is amongst the 20 most abundant proteins in the eukaryotic cell (Ghaemmaghami *et al*. 2003) and in response to thermal stress and other environmental stimuli, Hsp90 levels increase further (Taipale, Jarosz and Lindquist 2010). Lack of Hsp90 results in severe susceptibility to elevated temperatures (Borkovich *et al*. 1989). Under both non-stress and stress conditions, the chaperone functions by stabilizing a specific set of proteins, called target proteins or clients. Hsp90 forms a complex with its client until the client reaches its cellular destination or is required in its active state. At this point, the chaperone cycle is completed, and the mature client released (Zuehlke and Johnson 2010). Hsp90 is involved in numerous fundamental processes such signal transduction, cell growth and cellular differentiation in the eukaryotic cell (Zhao *et al*. 2005; Taipale *et al*. 2012).

Beyond functioning as a traditional chaperone, Hsp90 acts as an evolutionary capacitor. As such, it facilitates the storage and release of genetic variation. Under non-stress conditions, Hsp90 correctly folds its clients, masking any mutations their DNA sequences may accumulate because Hsp90 recognizes the 3D-structure of partially folded proteins rather than their sequence. Depleting Hsp90 either genetically or pharmacologically, however, removes the evolutionary buffer and causes clients to fold based solely on their amino acid sequence, causing the mutations to be expressed. Consequently, aberrations in plant (Queitsch, Sangster and Lindquist 2002) and fly (Rutherford and Lindquist 1998) morphology, as well as eye size in cave fish (Rohner *et al*. 2013) can be detected. In fungi, reducing Hsp90 function abolishes antifungal drug resistance (Cowen and Lindquist 2005). Hsp90’s ability to buffer a multitude of traits is due to its capacity to control expression of ∼20% of the pre-existing genetic variation (Jarosz and Lindquist 2010).

In addition to chaperoning client proteins, Hsp90 regulates gene expression in evolutionarily diverse organisms. For example, mammalian circadian clock genes (Schneider, Linka and Reinke 2014), plant phytohormone genes (Shigeta *et al*. 2015) and fungal stress-responsive kinase genes (Diezmann *et al*. 2012) require Hsp90 for expression. Due to Hsp90’s central role in fundamental cellular processes and its ability to shape evolutionary trajectories, it is critical to identify Hsp90 interactors. Elucidating Hsp90 interaction networks, of either direct or indirect nature, would allow for a comprehensive understanding of the protein complexes and cellular pathways Hsp90 is involved in. Yet, mapping Hsp90 networks in eukaryotes is technically challenging and requires extensive genetic and biochemical tools and techniques, such as genome-scale mutant libraries. Until recently these were only available in *S. cerevisiae*. Yet, with the development of molecular biology tools in other fungal species, Hsp90 interaction networks can now be mapped in a diverse range of fungi.

### Fungi represent an opportunity to study Hsp90 function and interaction networks in an evolutionary context

**Figure 1.**
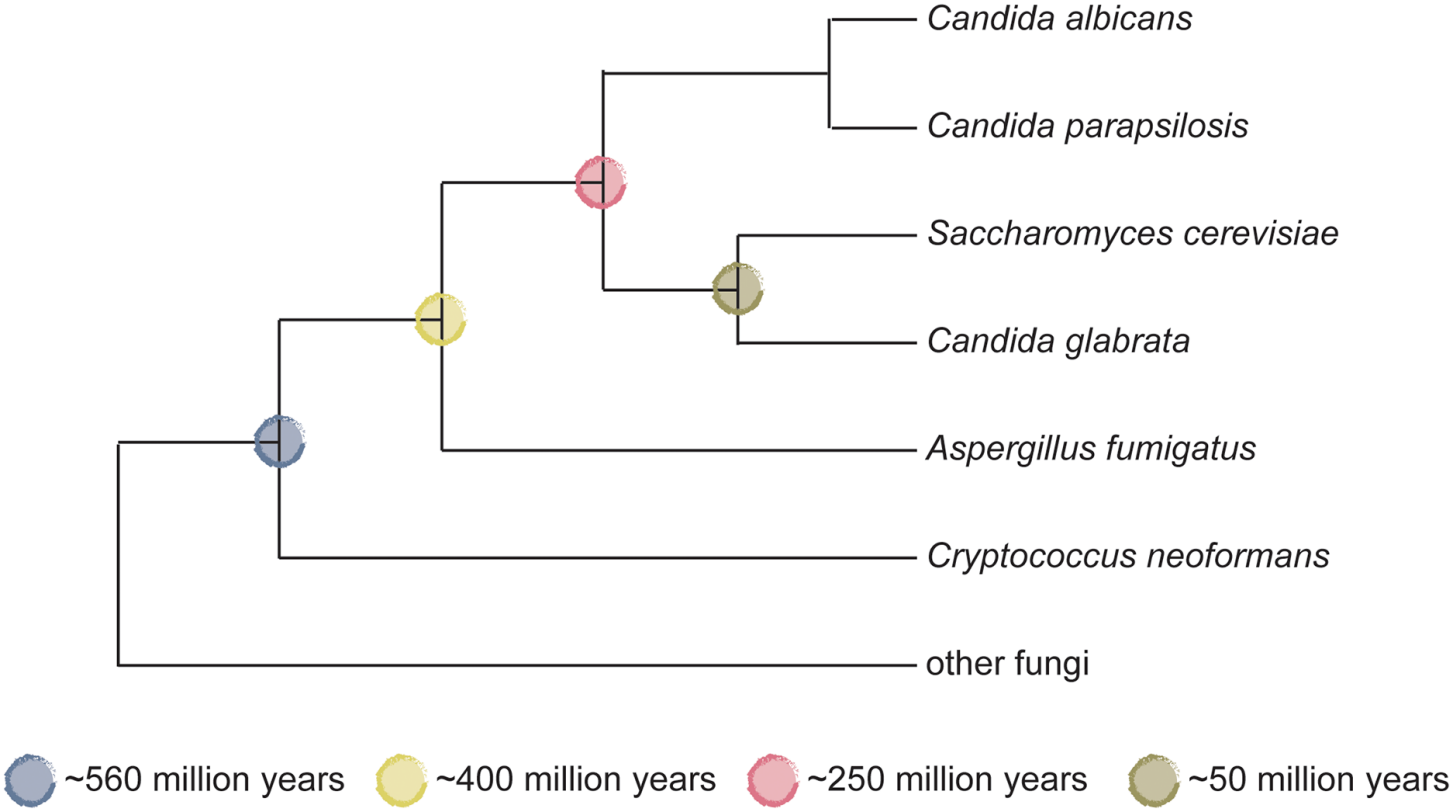
Phylogenetic relationships amongst fungal species with available mutant libraries. Divergence times for branches leading to *C. albicans*, *S. cerevisiae* and *C. glabrata* (all Saccharomycotina), *A. fumigatus* (Pezizomycotina) and *C. neoformans* (Basidiomycota) are indicated by colored circles.

Fungi are ancient. Fungal microfossils from the Canadian Northwest Territories date back to ∼1 billion years (Loron *et al*. 2019). Thus, fungi evolved before land plants, which emerged ∼470 million years ago on the fossil record (Edwards *et al*. 2014). Fungal species for which tools required for Hsp90 network mapping exist, span >500 million years of evolution (Fig. 1). Being able to map Hsp90 interaction networks in multiple fungal species across evolutionary time scales allows for powerful comparisons of how Hsp90 networks evolved, their evolutionary trajectories and the impact of ecology on network evolution. Within the subphylum Saccharomycotina, *S. cerevisiae* and *Candida glabrata* diverged ∼50 million years ago. They split from the ancestor of *Candidaalbicans* and *Candida parapsilosis* ∼250 million years ago. The Saccharomycotina diverged from the Pezizomycotina, the sub-phylum containing moulds such as *Aspergillus fumigatus*, ∼400 million years ago and the Dikarya, the group formed by the Ascomycota and the Basidiomycota, emerged ∼560 million years ago (Beimforde *et al*. 2014; Shen *et al*. 2020). Mutant libraries, required for Hsp90 genetic interaction network mapping, have been engineered in *C. albicans*, *C. parapsilosis*, *C. glabrata*, *A. fumigatus* and the basidiomycete *Cryptococcus neoformans* with the aim to comprehensively identify and study virulence factors. They also provide the unique opportunity to study Hsp90 network evolution.

### Hsp90 in *S. cerevisiae*

*Saccharomyces cerevisiae* has been instrumental in understanding eukaryotic Hsp90 function and regulation. Unlike other fungi, *S. cerevisiae* expresses two isoforms of Hsp90. One, Hsc82, is constitutively expressed at very high levels, and the other, Hsp82, is expressed at much lower levels but is strongly induced in response to heat shock. Expression of either gene is essential for growth, however, each isoform has distinct functions and clients (Borkovich *et al*. 1989; Girstmair *et al*. 2019). Crystalizing full-length *S. cerevisiae* Hsp90 together with an ATP analogue and a co-chaperone revealed the complex architecture of the clamp-like structure of the chaperone and the intricate conformational changes required to execute chaperone function (Ali *et al*. 2006). Due to its ease of manipulation, *S. cerevisiae* has been extensively used to identify and characterize the role of post-translational modifications in chaperone regulation. For more specific examples see this review by Mollapour and Neckers (2012). The phosphorylation site essential for survival of high temperatures was initially detected and characterized in *S. cerevisiae* (Nathan and Lindquist 1995).

### Hsp90 in *C. albicans*

Amongst pathogenic fungi, Hsp90 is best understood in *C. albicans*. This yeast causes ∼750 000 life-threatening invasive infections world-wide each year with mortality rates of up to 75% (Brown *et al*. 2012; Bongomin *et al*. 2017). In addition to this burden on human life and health, candidemia adds substantially to health care costs. The United States alone spent $1.4 billion on over 26 K hospitalizations necessitated by candidemia in 10 years (Benedict *et al*. 2019). High mortality rates and extended hospital stays are due to the currently available treatments being rather ineffective or the emergence of antifungal drug resistance. It is, thus, imperative to understand *C. albicans* virulence mechanisms and stress response pathways to develop more efficacious treatment strategies. Hsp90 is a key regulator of *C. albicans* virulence (Cowen *et al*. 2009), morphogenesis (Shapiro *et al*. 2009), drug resistance in planktonic cells (Singh *et al*. 2009) and biofilms (Robbins *et al*. 2011) as well as cell cycle progression (Senn, Shapiro and Cowen 2012). Despite promising results in insect models of fungal disease, targeting Hsp90 with inhibitors tested in clinical trials as anticancer drugs resulted in severe host toxicity in mice with candidemia. Due to the high sequence conservation between fungal Hsp90 and the mammalian ortholog, both othologs were inhibited, which caused complications for the host (Cowen *et al*. 2009). Hsp90 itself is therefore not suitable as an antifungal drug target until fungal-specific inhibitors of Hsp90 exist. Hence, alternative strategies need to be explored and Hsp90 interactors could prove useful as future drug targets. They could either be targeted by monotherapy or in combination therapy with already available Hsp90 inhibitors.

### Hsp90 in *C. parapsilosis*

*Candida albicans*’ relative *C. parapsilosis* causes 33% of candidemia infections in pre-term infants with mortality rates of 10% (Pammi *et al*. 2013). *Candida parapsilosis* infections can be difficult to treat due to reduced susceptibility to the echinocandin class of antifungals. This is caused by a naturally occurring amino acid substitution in the protein encoding the echinocandin target Fks1 relative to other *Candida* species (Garcia-Effron *et al*. 2008). Similar to *C. albicans*, Hsp90 represses filamentation in *C. parapsilosis* (Hossain, Veri and Cowen 2020) and the combination of triazoles and the Hsp90 inhibitor, geldanamycin, acts synergistically, reducing minimum inhibitory concentrations to triazoles (Mahmoudi *et al*. 2019). Beyond this, Hsp90’s role in virulence in this pathogen of premature infants remains uncharacterized.

### Hsp90 in *C. glabrata*

Although closely related to *S. cerevisiae*, comparatively little is known about Hsp90 in *C. glabrata*. This yeast is the leading cause of non-*albicans* candidiasis in Northern Europe and the United States of America, and the third or fourth in Asia (Kumar *et al*. 2019). Commensal strains found in the oral cavity or gut are often the causative agents in clinical *C. glabrata* infections (Pfaller *et al*. 2002; Wang *et al*. 2013; Guinea 2014; Khatib *et al*. 2016; Nash *et al*. 2017). *Candida glabrata* employs a suite of virulence traits that facilitate infection of humans including surface adhesion, biofilm production, tissue invasion, macrophage survival, immune dampening and drug resistance. Echinocandin resistance requires the environmentally responsive phosphatase, calcineurin, in *C. glabrata*, as in *C. albicans*. Inhibition or genetic repression of Hsp90 phenocopies that of Cnb1, a subunit of calcineurin, when measuring echinocandin resistance in *C. glabrata* (Singh-Babak *et al*. 2012). In addition to rapid development of drug resistance, *C. glabrata* is more intrinsically resistant to many drugs, especially the azole anti-fungals. Fluconazole became fungicidal instead of fungistatic when *C. glabrata* Hsp90 was inhibited by geldanamycin (Borah, Shivarathri and Kaur 2011). Apart from these studies, the role of Hsp90 in *C. glabrata* virulence remains unstudied.

### Hsp90 in *A. fumigatus*

The Pezizomycotina are a sister group to the Saccharomycotina yeasts. Amongst the Pezizomycotina, the ‘deadly mould’ *A. fumigatus* causes >300 000 invasive infections world-wide each year with mortality rates of up to 95% (Brown *et al*. 2012; Bongomin *et al*. 2017). In addition to causing life-threatening invasive infections, mainly of the lung, *A. fumigatus* causes chronic and allergic pulmonary disease in ∼8 million patients world-wide (Bongomin *et al*. 2017). The United States alone spent ∼$1.2 billion on hospitalizations necessitated by invasive aspergillosis over 10 years (Benedict *et al*. 2019). These staggering numbers are due to a suite of host- and fungal-specific factors. *Aspergillus fumigatus* is highly prevalent in the environmental and its small spore size allows the fungus to reach the bronchoalveolar space. Once inside the host, *A. fumigatus* effectively adheres to the human lung lumen and extensive secretion of galactosaminogalactan and extracellular proteases facilitate persistence in the human lung (Gago, Denning and Bowyer 2019). This is further confounded by a broad and increasing patient demographic that includes leukemic patients and those that received hematopoietic stem cell or solid organ transplants (Kontoyiennis *et al*. 2010; Pappas *et al*. 2010). Hsp90’s role in *A. fumigatus* virulence, drug resistance and morphogenesis has been reviewed here (Lamoth, Juvvadi and Steinbach 2016). More recently, Hsp90 expression, controlled by the transcription factor (TF) HsfA (Fabri *et al*. 2021), has been shown to be up-regulated in response to heat-shock and azole treatment (Tu, Yin and Li 2020). Resembling findings in *C. albicans* (Lafayette *et al*. 2010; Caplan *et al*. 2018), *A. fumigatus* Hsp90 governs the cell wall integrity pathway (CWIP) by stabilizing key kinases of this pathway (Rocha *et al*. 2021). While specific Hsp90 interactors, such as the CWIP kinases have been identified, a global view of Hsp90 genetic and physical interactors is yet to be obtained.

### Hsp90 in *C. neoformans*

The basidiomycetous yeast *C. neoformans*, the causative agent of the AIDS-defining illness cryptococcal meningitis, causes >1 million cases world-wide each year with mortality rates of up to 70% (Brown *et al*. 2012). From its environmental reservoirs, mainly pigeon guano but also Eucalyptus trees (Edwards *et al*. 2021), *C. neoformans* spores enter the host via inhalation and then move to the brain (Lin and Heitman 2006). Important virulence factors supporting host colonization include an antiphagocytic capsule (Kozel *et al*. 1988) and melanin production, which provides protection from UV light, oxidative stress, microbicidal peptides and phagocytic cells (Casadevall, Steenbergen and Nosanchuk 2003). Upon entry of *C. neoformans* into the mammalian lung, Hsp90 is up-regulated (Hu *et al*. 2008). Pharmacological inhibition of Hsp90 reduced *C. neoformans* tolerance to thermal stress, antifungal drugs and virulence in an invertebrate model of fungal virulence (Cordeiro *et al*. 2016). It has, furthermore, been observed that Hsp90 is physically associated with the *C. neoformans* cell wall and regulates capsule induction and maintenance (Chatterjee and Tatu 2017). Hsp90 interactors are yet to be identified and characterized in *C. neoformans*.

To date, ten fungal Hsp90 interaction networks have been mapped. Due to limited availability of essential tools, such as genome-scale mutant libraries, Hsp90 networks are currently restricted to the eukaryotic model system *S. cerevisiae* and the major fungal pathogen *C. albicans*. Here, we will review the tools and technologies that made Hsp90 network mapping possible and how they may be extended into other fungal species.

### Mutant libraries available in fungi

To get a global view of the genes and proteins that either directly or indirectly interact with Hsp90, a suite of genetic and molecular biological manipulations is required. These include genome-scale collections of loss-of-function mutants and epitope-tagged strains.

**Figure 2.**
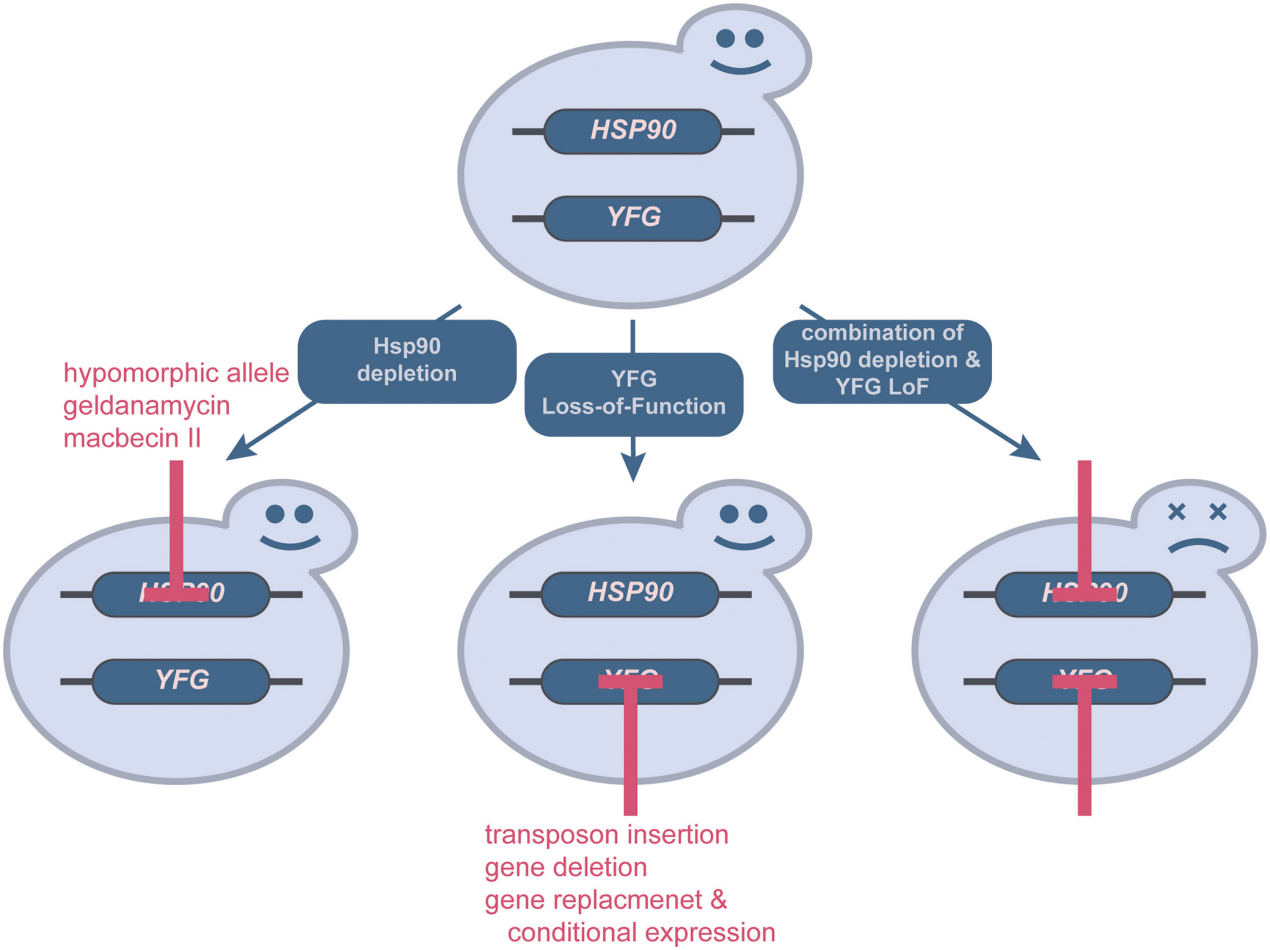
Synthetic lethality identifies genes acting in the same pathway or complex. Yeast cells are viable when experiencing either sub-lethal depletion of Hsp90 function or loss of function of ‘your favourite gene’ (YFG). The combination of both, however, is not tolerated and yeast cells are either ‘sick’ (reduced growth) or dead. Hsp90 function can be reduced by either pharmacological inhibition or the use of hypomorphic alleles and loss-of-function mutations can be achieved as described in the text.

Genetic interactions are defined as two genes that together produce an unexpected phenotype (Costanzo *et al*. 2019; Fig. 2). To map genome-scale Hsp90 genetic interaction networks, suitable mutant collections are required. These mutant libraries, containing hundreds or thousands of loss-of-function mutants, were initially developed to study eukaryotic gene function in *S. cerevisiae* or to identify virulence genes in pathogenic fungi. To generate these collections (Table 1), the majority of which were made possible by community efforts, different genetic approaches were deployed.

**Table 1.** Fungal mutant libraries.

Species	# Mutants/# genes	Background strain	Mutant auxotrophic and resistance markers	Reference	Availability
*S. cerevisiae*	4815/4815 MATa barcoded haploid deletionmutants	BY4730	leu, met, ura, kan^R^	Giaever *et al*. (2002)	Horizon Discovery Ltd.(https://bit.ly/3xqwJTR)
*S. cerevisiae*	4803/4803 MATalpha barcoded haploid deletion mutants^1^	BY4739	leu, lys, ura, kan^R^	Giaever *et al*. (2002)	Horizon Discovery Ltd.(https://bit.ly/3xqwJTR)
*S. cerevisiae*	4757/4757 barcoded homozygous diploid mutants^2^	BY4743	his, leu, ura, kan^R^	Giaever *et al*. (2002)	Horizon Discovery Ltd.(https://bit.ly/3xqwJTR)
*C. albicans*	1248/703 transposon insertion (Tn7) mutants of transcription factor, kinase and random genes	BWP17(Wilson, Davis and Mitchell 1999)	his	Davis *et al*. (2002)	Fungal Genetics Stock Centre (https://bit.ly/3xqmI8Z)
*C. albicans*	365/166 transcriptional regulator knockouts	SN152 (SC5314)(Noble and Johnson 2005)	arg	Homann *et al*. (2009)	Fungal Genetics Stock Centre (https://bit.ly/3xqmI8Z)
*C. albicans*	3000/674 barcoded gene deletions	SN152 (SC5314; Noble and Johnson 2005)	arg	Noble *et al*. (2010)	Fungal Genetics Stock Centre (https://bit.ly/3xqmI8Z)
*C. albicans*	2357/2357 Gene Replacement and Conditional Expression (GRACE) mutants (Merck-Frosst library)^3^	CaSS1 (CAI4)	nat^R^	Roemer *et al*. (2003)	National Research Council of Canada (https://bit.ly/3yyPX9J)
*C. albicans*	5099 ORF clones using Invitrogen Gateway technology (ORFeome collection for C2H)	pDONR207 (Brand, MacCallum and Walker 2012)		Legrand *et al*. (2018)	http://candidaorfeome.eu/ https://www6.inrae.fr/cirm_eng/
*C. parapsilosis*	200/100 barcoded gene deletions of transcription factors, kinases, species-specific genes	CLIB214		Holland *et al*. (2014)	Please contact the authors
*C. glabrata*	1601/619 barcoded gene deletions	HTL	natR, his, trp, leu	Schwarzmüller *et al*. (2014)	Please contact the authors
*A. fumigatus*	484/484 transcription factor null mutants	MFIG001 (A1160; Fraczek *et al*. 2013)	ku80-, pyrG+, hphR	Furukawa *et al*. (2020)	National Collection of Pathogenic Fungi(https://bit.ly/3AxPCWH)
*C. neoformans*	322/155 barcoded transcription factor deletion mutants	H99S	natR	Jung *et al*. (2015)	Fungal Genetics Stock Centre (http://www.fgsc.net/crypto/crypto.htm)
*C. neoformans*	264/129 barcoded kinase deletion mutants	H99S	natR	Lee *et al*. (2016)	Fungal Genetics Stock Centre (http://www.fgsc.net/crypto/crypto.htm)
*C. neoformans*	230/114 barcoded phosphatase deletion mutants	H99S	natR	Jin *et al*. (2020)	Fungal Genetics Stock Centre (http://www.fgsc.net/crypto/crypto.htm)
*C. neoformans*	2112/2112 barcoded gene deletion mutants (2015 set)	KN99alpha (Nielsen *et al*. 2003)	natR	Chun and Madhani (2010)	Fungal Genetics Stock Centre (http://www.fgsc.net/crypto/crypto.htm)
*C. neoformans*	1919/1919 barcoded gene deletion mutants (2016 set)	KN99alpha (Nielsen *et al*. 2003)	natR	Chun and Madhani (2010)	Fungal Genetics Stock Centre (http://www.fgsc.net/crypto/crypto.htm)
*C. neoformans*	662/662 barcoded gene deletion mutants (2020 set)	KN99alpha (Nielsen *et al*. 2003)	natR	Chun and Madhani (2010)	Fungal Genetics Stock Centre (http://www.fgsc.net/crypto/crypto.htm)

1 Barcoded haploid MATa and MATalpha deletion libraries in the BY4741 (MATa, *his3*∆, *leu2*∆, *met15*∆, *ura3*∆ and*KanMX*) and BY4742 (MATalpha, *his3*∆, *leu2*∆, *lys2*∆ and*ura3*∆) are also available.

2 A barcoded heterozygous diploid mutant library in the BY4743 background containing 5916 mutants exists as well.

3 Merck Sharp and Dohme Corp has also produced a heterozygous double barcoded library with 5467 mutants (Xu *et al*. 2007). This library is available from the National Research Council of Canada (https://bit.ly/3yyPX9J).

Gene deletion libraries are easier to assemble in haploid organisms, as only one allele requires manipulation. *Cryptococcus neoformans, C. glabrata* and *A. fumigatus* are haploid, while *C. albicans* and *C. parapsilosis* are diploid. In contrast, non-laboratory *S. cerevisiae* strains are usually diploid (Diezmann and Dietrich 2009), while the *S. cerevisiae* laboratory strain S288c is an artificial haploid (Mortimer and Johnston 1986). This is due to mutations in the HO-endonuclease, whose wild-type facilitates mating-type switching and consequently selfing (Meiron, Nahon and Raveh 1995).

### *Saccharomyces cerevisiae* mutant libraries

*Saccharomyces cerevisiae* has been a trailblazer in building interaction networks due to ease of genetic manipulation and a readily executable sexual cycle. Various libraries, differing by mating type, ploidy and auxotrophic markers have been generated (Table 1). To delete target genes in a haploid background, wild-type alleles of non-essential genes were replaced with the *KanMX* cassette, conferring resistance to the antibiotic geneticin (G418) to select for successful transformation events. These haploid deletion strains were then mated and selected for based on their auxotrophies to create diploid homozygous deletion strains (Giaever *et al*. 2002). Additionally, in these libraries the KanMX cassette is flanked 3’ and 5’ by 20-mer oligonucleotides, which serve as unique barcodes. Barcode frequencies can either be quantified via microarray technology or next-generation sequencing, allowing pooling of libraries and screening of thousands of mutants in a single vial under the exact same conditions.

### *Candida albicans* mutant libraries

Research on pathogenic fungi is often hampered by low homologous integration rates and the absence of a canonical sexual cycle. Consequently, different strategies have been implemented to manipulate gene function. The situation is further confounded by *C. albicans* being diploid, which means that depletion of gene function requires two rounds of transformation. This is usually done using a modified *S. cerevisiae* lithium acetate protocol, which can inadvertently lead to changes in chromosome copy numbers (Bouchonville *et al*. 2009).

The first publicly available mutant library deployed random transposon insertion mutagenesis (Davis *et al*. 2002). Transposon mutagenesis is a widely used technique through which genetic material, such as auxotrophic markers, are distributed throughout the host genome via a mobile DNA element, the transposon. This process is mostly random but can lead to gene inactivation, should the transposon insert into coding DNA. Here, the Tn7 transposon, carrying the *UAU1* cassette (*Tn7-UAU1*), was transformed into the *C. albicans* genome. The *UAU1* cassette contains a complete copy of *ARG4* flanked by the 3’ and the 5’ regions of *URA3*. Transforming the *Tn7-UAU1* construct into *C. albicans* strain BWP17 (arg, his and ura) yields Arg+ transformants upon successful integration into allele one. Should recombination of the *UAU1* cassette occur, the *URA3* marker reconstitutes while being inserted into allele two, therefore, homozygous mutants are Arg+ Ura+. This strategy delivered >1200 histidine–auxotroph transposon insertion mutants representing 703 genes. It should be noted that the progenitor strain used to generate this library, BWP17, is missing a part of the right arm of chromosome 5B (Forche *et al*. 2004; Selmecki, Bergmann and Berman 2005)

Conversely, a TF library was produced using a clean gene deletion approach to remove both alleles of 166 non-essential TF genes (Homann *et al*. 2009). Auxotrophic markers *HIS1* and *LEU2* replace each wild-type TF allele in the progenitor strain SN152 (Noble and Johnson 2005). This approach was then expanded to create a homozygous gene deletion library containing 3000 mutants representing 674 genes (Noble *et al*. 2010). These mutants are also tagged with one of 48 different oligonucleotide barcodes. This 20-mer, adjacent to the selectable marker, allows for mutants to be pooled in groups of 48, thereby drastically reducing the experimental load.

In addition to transposon insertions and clean gene deletions, *C. albicans* loss-of-function mutants have also been created using a gene replacement and conditional expression (GRACE) strategy (Roemer *et al*. 2003). To produce each mutant, the progenitor strain CaSS1, a histidine–auxotroph CAI derivative, has one allele replaced with the *HIS3* auxotrophic marker, flanked by two distinct barcodes, and the second allele's promoter is replaced by a *SAT1*-marked tetracycline promoter. Transformants were then selected for nourseothricin resistance and expression of allele two can be repressed by culturing with doxycycline or tetracycline. It was successfully used to identify essential genes in *C. albicans*. The library design allows for the prototrophic and nourseothricin resistant mutants to be pooled but limits further genetic manipulations.

**Figure 3.**
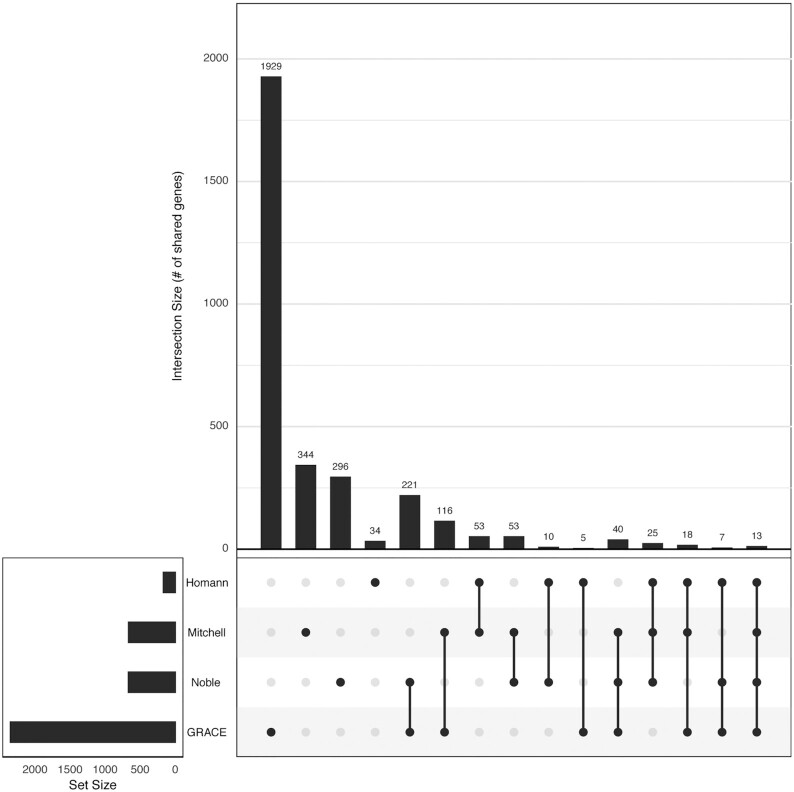
*Candida albicans* mutant libraries sizes and overlaps. Upset *R*-plot depicting the size of each library on the left (set size) and the overlap between different libraries on the right. The Homann library covers 166 TF gene deletions (Homann *et al*. 2009). The Mitchell library consists of 703 genes disrupted by transposon insertions (Davis *et al*. 2002). The Noble library comprises 674 clean gene deletion mutants (Noble *et al*. 2010). The GRACE library provides repressible mutants for 2357 genes (Roemer *et al*. 2003). Vertical bars represent the number of genes shared between each of the libraries, the libraries sharing these genes are indicated by the connected dots. There is little overlap in genes represented between libraries, together these libraries allow disruption of 2603 genes, covering 42% of the *C. albicans* genome.

Of the four *C. albicans* libraries described above, there is very little overlap in genes represented (Fig. 3). The largest portion of genes, 221, is shared by the Noble and the GRACE library. Only 13 genes are covered by all four libraries. Thanks to these efforts, the *C. albicans* community has access to four mutant libraries that together cover ∼50% of the genome.

### *Candida parapsilosis* and *C. glabrata* libraries

Homologous integration rates are also extremely low in the other *Candida* species, including *C. parapsilosis* and *C. glabrata*. To counter-act this and improve transformation rates, wild-type alleles were replaced in both species with constructs flanked by 500 bp homology arms made using fusion PCR (Noble and Johnson 2005). To create the *C. parapsilosis* library, the first wild-type allele was replaced with the *C. maltosa LEU2* gene and the second allele with the *C. dubliniensis HIS1* gene (Holland *et al*. 2014). Each of the 200 mutants, two per gene, is also barcoded with a 20-mer signature DNA tag permitting pooling of otherwise prototrophic mutants. To replace wild-type alleles in haploid *C. glabrata*, the *NAT1* marker (Shen, Guo and Köhler 2005), conferring resistance to nourseothricin, flanked by two barcodes, was deployed. The library, containing 1601 mutants, covers ∼10% of the genome and mutants can be pooled in groups of 96 (Schwarzmüller *et al*. 2014). Mutants are nourseothricin resistant and auxotrophic for histidine only or for histidine, tryptophan and leucine, permitting further manipulations if required.

### The *A. fumigatus* library

To create the *A. fumigatus* gene deletion library, the hygromycin B phosphotransferase cassette (hph) was amplified with 1 kb flanking regions using a fusion PCR protocol (Szewczyk *et al*. 2006). This construct was transformed into the progenitor strain MFIG001 (Fraczek *et al*. 2013). MFIG001 is deficient for homologous end joining (akuB^KU80^; Kress *et al*. 2006) and carries the pyrG gene, which encodes the orotidine-5’-phosphate decarboxylase, complementing for uracil and uridine auxotrophy (Osmani, Oakley and Osmani 2006). As a consequence, the almost 500 TF gene deletion mutants are prototrophic and resistant to hygromycin B, precluding further genetic modifications (Furukawa *et al*. 2020).

Different sets of mutant libraries exist for *C. neoformans*. In a targeted approach utilizing database predictions of gene function, wild-type alleles of TFs, kinases and phosphatases were deleted (Jung *et al*. 2015; Lee *et al*. 2016; Jin *et al*. 2020) in the wild-type strain *C. neoformans* H99S (Janbon *et al*. 2014). To do so, the nourseothricin-resistance marker *NAT1* was amplified together with a barcode using either an overlap PCR (Davidson *et al*. 2002) or a double-joint PCR approach (Kim *et al*. 2009) and transformed into H99S. Mutants, usually multiple per gene, are prototrophic and barcoded for ease of handling. A second, much larger, set of *C. neoformans* mutants was generated by transforming wild-type strain KN99alpha with overlap fusion PCR products (Chun, Liu and Madhani 2007). The transformation constructs harbor the *NAT1* marker, together with 48 unique barcodes (Liu *et al*. 2008) flanked by 1 kb regions of homology to the up- and down-stream regions of the target gene. In this library, each gene is represented by one prototrophic, nourseothricin resistant, barcoded mutant. Note that H99S and KN99 are derivatives of H99, which was collected from a patient at Duke Medical Center in 1978. For genetic relationships amongst these and other H99 derivatives see here (Janbon *et al*. 2014).

Together, these mutant libraries enable the large-scale studies into genetic interactions, including that of Hsp90 across evolutionary time in species with different life history trajectories.

### Useful techniques for mapping Hsp90 networks in fungi

Several genetic and biochemical approaches have been developed to map Hsp90 interaction networks. Screens are conducted to catalog genes or proteins that are part of the same cellular pathways as Hsp90 or depend on Hsp90 for regulation, stability and/or activation.

### Perturbing Hsp90 function

Knock-out mutants have long provided a fundamental technique to investigate the function and interactions of genes, however, this is confined only to non-essential genes. Since Hsp90 is essential, deletion mutants are not viable, so pharmacological inhibition or genetic depletion have been used to carry out synthetic lethality screens to build Hsp90 interaction networks. Pharmacological inhibition of Hsp90 can be achieved by addition of one of several commercially available Hsp90 inhibitors, such as geldanamycin and macbecin II, which would be the ones most commonly used in Hsp90 screens. It should be noted that while both drugs are commonly considered to inhibit Hsp90 by binding to its ATP-binding pocket (Prodromou *et al*. 1997; Martin *et al*. 2008), they appear to exert multiple subtle effects that in combination reduce Hsp90 function and may cause small off-target effects (Schmid, Götz and Hugel 2018). Genetic depletion of Hsp90 is possible by producing mutants where Hsp90 is under a repressible promoter such as the tetracycline promoter (Gari *et al*. 1997; Nakayama *et al*. 2000). Both, chemical and genetic Hsp90 perturbation, allow fine tuning of Hsp90 function to elicit synthetic lethality in loss-of-function libraries, a prerequisite for the mapping of genetic interaction networks.

### Synthetic lethality screens

Genetic interactions occur between genes in the same pathways or molecular complexes, and the functional associations between genes throughout the genome form a genetic interaction network. Genetic interactions can be investigated using the concept of synthetic lethality. Synthetic lethality states that if two non-essential genes genetically interact, the viability of a double mutant is significantly affected, causing reduced fitness (synthetic sickness) or death (synthetic lethality; Dobzhansky 1946; Bendert and Pringle 1991). Organisms have inherent redundancy in their cellular pathways, thereby if one component is not functional, a second route exists that can by-pass the non-functional component. However, when two interacting genes are not functioning, the pathway is no longer functional, and the organism's survival is affected (Fig. 2). Gene deletion libraries can be screened for inviability in response to Hsp90 inhibition in studies termed chemical genetic synthetic lethality (CGSL) screens.

Synthetic genetic arrays (SGA) also use the premise of synthetic lethality but exploit sexual recombination to cross haploid *S. cerevisiae* gene deletion strains (Tong *et al*. 2001). Again, since Hsp90 is essential, a hypomorphic Hsp90 allele must be used. Inviable crosses indicate that the non-functional genes in the haploid parents genetically interact (Novick, Osmond and Botstein 1989).

### Y2H and C2H protein–protein interaction screens

Hsp90 forms numerous protein complexes with clients and co-chaperones through its role as a molecular chaperone. Some co-chaperones contain a conserved Hsp90-binding sequence, however, client proteins lack such a motif (Scheufler *et al*. 2000). This hinders bioinformatic prediction of Hsp90 clients, necessitating proteomic and physical interaction screens.

**Figure 4.**
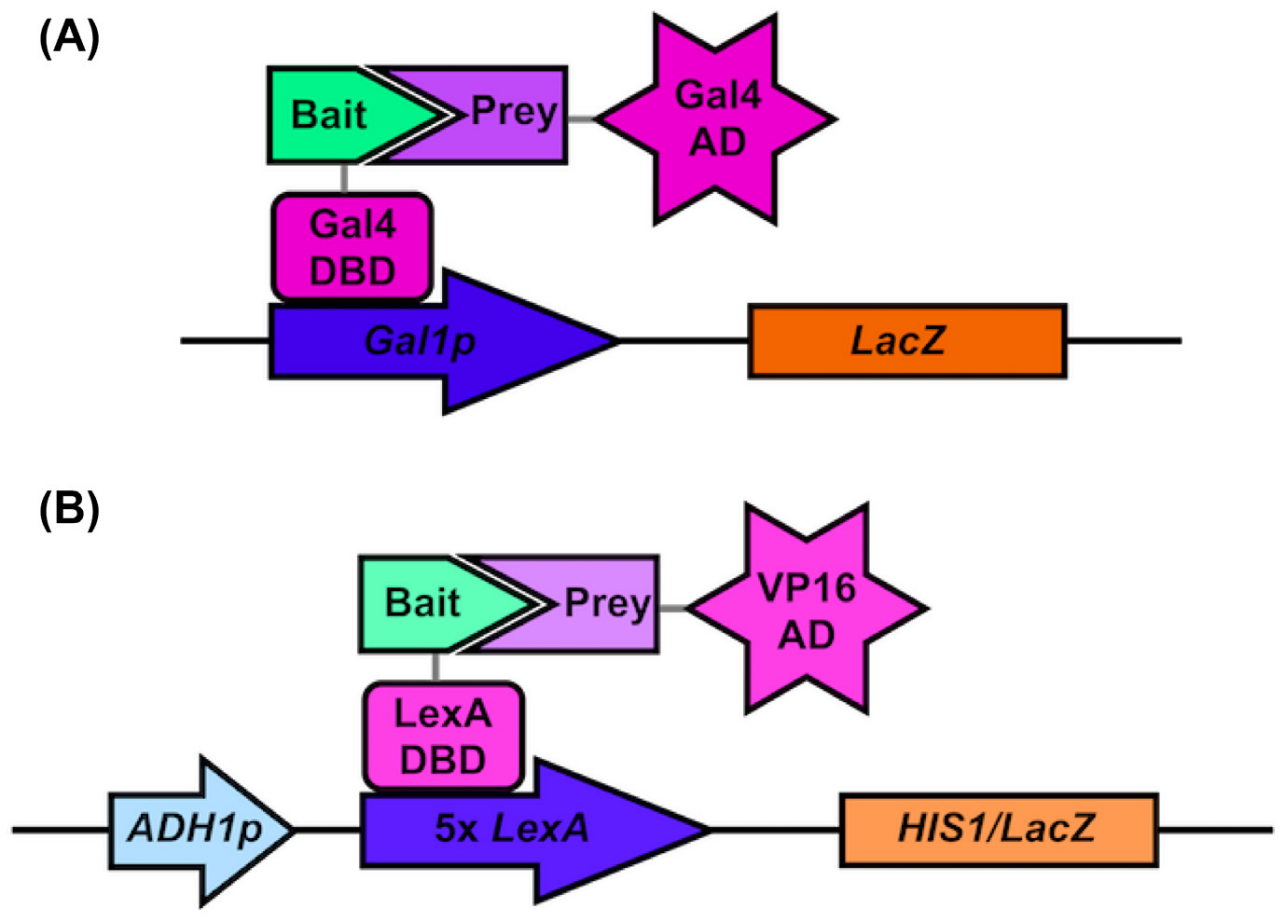
Set-up of the original yeast two-hybrid system for use in *S. cerevisiae* and its adaption to *Candida* two-hybrid. **(A)** The original yeast two-hybrid system uses *LacZ* as the reporter gene (Fields and Song 1989). When the Gal4 DBD-tagged bait protein interacts with the Gal4 AD-tagged prey protein, Gal4 induces the expression of *LacZ* via the *GAL1* promoter. Colonies where bait and prey proteins interact will appear blue when grown on X-gal media. **(B)** The *Candida* two-hybrid system uses *C. albicans* optimized genes (Stynen, van Dijck and Tournu 2010; Legrand *et al*. 2018; Schoeters *et al*. 2018). The background strain, SC2H3, has two reporter genes, *Streptococcus thermophilus LacZ* and *C. albicans HIS1*. Each reporter gene is under the *C. albicans ADH1* promoter and five copies of the *Staphylococcus aureus LexA* operon. The *LacZ* reporter cassette is integrated into chromosome 1 and the *HIS1* reporter cassette is integrated into chromosome 4. When *S. aureus LexA* DBD-tagged bait interacts with viral VP16 AD-tagged prey, expression of *LacZ* and *HIS1* is induced. Strains where bait and prey proteins interact will grow on histidine deficient media and have increased β-galactosidase activity, measurable via assay.

A canonical, large-scale method to identify protein–protein interactions (PPIs) is the yeast two-hybrid system (Y2H; Fields and Song 1989). This system employs a reporter gene such as *HIS3*, *ADE2*, *LEU2* or *Escherichia coli LacZ* down-stream of an inducible promoter such as *GAL1p* (Vojtek, Hollenberg and Cooper 1993; Estojak, Brent and Golemis 1995; James, Halladay and Craig 1996). The original and most utilized system uses the *S. cerevisiae* TF, Gal4 to bind to *GAL1p* and induce expression of *LacZ*. Gal4 comprises an N-terminal DNA binding domain (DBD) and a C-terminal activator domain (AD). When both Gal4 domains come together, expression of β-galactosidase from *LacZ* is induced. On addition of 5-bromo-4-chloro-3-indolyl-β-D-galactopyranoside (X-gal), β-galactosidase cleaves the X-gal to form a blue-colored molecule. In Y2H screens the Gal4 binding domain is fused to one protein (termed the ‘bait’) and the activating domain to another protein (‘prey’). If these proteins interact, Gal4 is reconstituted, allowing expression of β-galactosidase and cleavage of X-gal, causing the colony to turn blue (Fig. 4A). To enable high-throughput screening of the *S. cerevisiae* proteome, a haploid *MATa* bait library containing the Gal4 DBD attached to each protein in the *S. cerevisiae* proteome can be mated with a *MATalpha* prey strain containing a gene of interest attached to the Gal4 activating domain (Uetz *et al*. 2000). When crossed, selected for diploid cells and grown with X-gal, blue colonies indicate that the Gal4-domain tagged proteins in the haploid parental strains directly and physically interact.

Due to *C. albicans’* alternative codon usage (CUG coding for leucine instead of serine) and this species’ inability to maintain plasmids, Y2H systems designed in *S. cerevisiae* are not reliable in this pathogenic yeast. The first 2H system designed for *C. albicans* (C2H) comprises plasmids that are integrated into the genome (Stynen, van Dijck and Tournu 2010). A total of two reporter genes, *C. albicans HIS1* and *Staphylococcus thermophilus LacZ* were used, up-stream of five copies of the *Staphylococcus aureus* LexA operon and the *C. albicans ADH1* promoter. An artificial TF comprised of *S. aureus LacZ* DBD and the viral activating domain VP16 activate the *LexA* operon and induce expression of *LacZ* and *HIS1*. The bait and prey plasmids allow the tagging of genes of interest with the *LexA* DBD and *VP16* activating domain under the *MET3* repressible promoter. Reporter gene, bait and prey plasmids are then linearized and integrated into sections of the *C. albicans* genome where their affects were predicted to be minimal. If the bait and prey proteins interact, strains grown in methionine deficient media are able to grow on histidine selection media and have high activity in a β-galactosidase assay (Fig. 4B).

This C2H system was developed by making the vector plasmids Gateway compatible and mating-inducible *C. albicans* strains were constructed to allow crossing of bait and prey strains rather than triple-transforming the same strain with three plasmids (Legrand *et al*. 2018). Again, further improvements to allow high-throughput screens were made by optimizing a mating protocol on agar rather than in broth (Schoeters *et al*. 2018). Furthermore, the ORFeome library (each *C. albicans* ORF in a Gateway vector, Table 1) allows the cloning of each *C. albicans* ORF into the bait or prey C2H vectors for high-throughput interaction studies. Now, the mating-inducible strain containing the reporter gene cassette can be transformed with either the bait or prey plasmids, mated and screened for histidine prototrophy, which indicates the bait and prey proteins interact.

### Tandem affinity purification—mass spectrometry proteomics

The Y2H and C2H systems involve several transformations, which is not an insignificant task on a high-throughput level, especially in *C. albicans* which has low rates of homologous integration. An alternative proteomic approach to investigate PPIs is that of tandem affinity purification (TAP) coupled with mass spectrometry (MS; Rigaut *et al*. 1999). The TAP tag is formed of calmodulin binding peptide (CBP) and protein A (ProtA) linked with a Tobacco Etch Virus protease (TEV) cleavage site. Tagging a protein with this epitope allows stringent purification first using ProtA's strong affinity to immunoglobulin G (IgG), the protein is then released by TEV protease cleavage and purified again using calmodulin in the presence of Ca^2+^. Chelating the calcium ions by egtazic acid (EGTA) releases the TAP-tagged protein from calmodulin. Any proteins that form stable complexes with the TAP-tagged protein will co-purify and can be identified by MS. This technique needs only one transformation of a yeast, tagging a gene of interest with the TAP epitope, of which *S. cerevisiae* and *C. albicans* optimized sequences exist (Gavin *et al*. 2002; Lavoie *et al*. 2008).

### Multiplexed quantitative proteomics

Another approach is to utilize multiplexed quantitative proteomics to identify both direct and indirect interactions of Hsp90. The most used technique, stable isotope labeling by amino acids in cell culture (SILAC), involves growing cells in the presence of heavy or light carbon-labeled amino acids, usually arginine and lysine, while inhibiting or repressing Hsp90 (Ong *et al*. 2002; Gopinath *et al*. 2014; O'Meara *et al*. 2019). The cells assimilate these isotopic amino acids into newly synthesized proteins. When subjected to MS, the differently labeled samples can be quantified individually, allowing comparisons between up to three samples at once: those with natural isotopes, light isotopes and heavy isotopes. A more powerful quantitative proteomic approach is Tandem Mass Tagging (TMT; Thompson *et al*. 2003). This technique covalently attaches one of up to ten different isobaric tags of the same mass to a cell protein sample. During MS, the tags fragment into reporter ions with differing masses, allowing comparison of up to 11 samples simultaneously. Since many samples can be compared concurrently and because tagging occurs after protein extraction, TMT allows robust quantitative proteomic studies without extremely high costs or specialist cell-culture techniques. Although TMT proteomics is yet to be applied to fungal Hsp90 studies, this technique has been used on Hsp90-inhibited human lung cancer and squamous cell carcinoma cell lines (Grimes *et al*. 2018; Mehta *et al*. 2020).

Quantitative proteomics on Hsp90-impaired cells can identify interactors up and down-stream of Hsp90 in molecular pathways. Interactors up-stream of Hsp90 are likely to increase in abundance to mitigate loss of Hsp90 function, while clients which are dependent upon Hsp90 for their stability and folding will decrease in abundance. Since clients lack a conserved Hsp90 binding motif to allow identification bioinformatically, quantitative proteomics provides a unique, proteome-wide view to predict novel Hsp90 clients.

The use of these techniques provides a comprehensive toolbox to investigate genetic, proteomic and PPIs.

### Fungal Hsp90 interaction networks

Inhibiting Hsp90 function results in a multitude of phenotypes indicative of the central role this molecular chaperone plays. Genetic, physical and proteomic interaction networks allow identification of the molecular pathways and complexes through which Hsp90 exerts its control. To date, ten networks have been mapped in two fungi, yielding insights into Hsp90 function and regulation (Table 2).

**Table 2.** Hsp90 interaction networks at a glance.

Organism	Experimental technique	# of interactors	Key discoveries	Reference
*S. cerevisiae*	Yeast two-hybrid (Y2H) using the Hsp82^E33A^ mutant as bait with a library of ∼6000 prey strains. The E33A allele stabilizes transient Hsp90 interactions.	177	Hsp82 interacts with ∼3% of the *S. cerevisiae* proteome, including Hsp60/Hsp10, cytoskeletal proteins and membrane transporter proteins. Characterization of MAPK Slt2p as a novel Hsp82 client. Stress-activated double phosphorylation of Slt2p (T190, Y192) essential for Hsp82–Slt2p interaction.	Millson *et al*. (2005)
*S. cerevisiae*	Y2H using different Hsp82 domains and full-length Hsp82 as bait against the entire *S. cerevisiae* genome.TAP-MS using N-terminal tagged Hsp82 and ∼4000 C-terminal TAP-tagged single gene constructs.Synthetic Genetic Array (SGA) on a *MATα hsc82∆ HSP82^ts^* strain mated with the *MATa* deletion library of ∼4700 non-essential genes. Nonviable or slow growing diploids at 35°C indicative of Hsp90 genetic interaction.Chemical genetic synthetic lethality (CGSL) screen of barcoded haploid deletion library grown in the presence of Hsp90 inhibitor geldanamycin (GdA).	Y2H = 90TAP-MS = 118SGA = 300CGSL = 200Total = 627	Hsp90 is a network hub interacting with at least 10% of the proteome. Comparison of physical and genetic interaction networks. Hsp90 interacts with genes with a diverse range of functions, including cellular fate/organization, cellular transport, metabolism, protein fate and transcription. Novel co-chaperones Tah1 and Pih1 interact with Rvb1/Rvb2 (components of chromatin remodeling factors), linking Hsp90 to epigenetic gene regulation.	Zhao *et al*. (2005)
*S. cerevisiae*	CGSL screen on a barcoded homozygous diploid deletion library using the Hsp90 inhibitor macbecin II at 30°C and 37°C.CGSL screen on barcoded heterozygous diploid deletion library at 30 and 37°C.	Homozygous screen:102 at 30°C118 at 37°C90 sharedTotal = 310Heterozygous screen:235 at 30°C241 at 37°C 40 sharedTotal = 516	Differences in Hsp90 interactions depending on culture conditions. Hsp90 interactors are enriched for hub proteins with at least 25 interactors. Hsp90 is essential for cell cycle progression, cytokinesis, trafficking of proteins to the vacuole, secretion. GO term enrichment in homozygous screen: kinases, mitochondrial membrane components, transcription factors, transport-related categories, secretory pathway and protein complex subunits at 30°C, microtubule organizing centre, signal transduction, cell-cycle, cytokinesis, bud components and metabolism including thermal stress response at 37°C. GO term enrichment in heterozygous screen: cellular transport.	McClellan *et al*. (2007)
*S. cerevisiae*	Mass-spectrometric identification of interactions between 63 TAP-tagged chaperones and 4562 TAP-tagged individual genes.	259 chaperone–chaperone interactions4340 chaperone–protein interactions	Numbers of non-chaperone interactions vary greatly between chaperones (e.g. 2 for Hsp32 and 3269 for Ssb1). Some proteins are specialists (bind to one chaperone only), others are promiscuous (bind to many chaperones). Identification of protein features that increase binding to chaperones (stretches of 1-5 hydrophobic residues, hydrophilic, larger/multi-domain, enriched for Asp, Glu and Lys, essential). The nucleus is a chaperone hot-spot while the further away from the nucleus a protein localizes to, the fewer chaperones it interacts with and proteins in the ER and mitochondria have fewer still.	Gong *et al*. (2009)
*S. cerevisiae*	CGSL screen on barcoded heterozygous diploid mutant library at 15°C.Combined analysis with the published heterozygous screens at 30 and 37°C (McClellan *et al*. 2007).	Total at 15°C = 27356 shared at 15 and 30˚C20 shared at 15 and 37˚C10 shared at all temperatures	Higheroverlap between similar temperatures. GO term enrichment: translation termination, translation elongation and protein targeting to membrane.	Franzosa *et al*. (2011)
*S. cerevisiae*	SILAC proteomics on *hsp82∆ TETp-HSC82* ura3::tTA strain coupled with transcriptomics.Combined analysis with publicly available data from *S. cerevisiae* and human.	Total = 90466% of interactors displayed no change in transcript levels. 74% of post-transcriptionally regulated proteins displayed decreased abundance, putative clients.	Misregulation of proteins by Hsp90 was mostly post-transcriptional. Part of the proteome that is regulated by Hsp90 is functionally conserved between yeast and human. Post-transcriptionally regulated proteins that decreased in abundance had a slower evolutionary rate providing evidence for Hsp90’s role as evolutionary capacitor. Depleted proteins enriched for essential genes and DNA repair proteins. Upregulated proteins enriched for stress response, protein folding and stabilization, unfolded protein response, mitochondrial electron transport, oxidative stress and metabolic processes.	Gopinath *et al*. (2014)
*C. albicans*	CGSL screen on transposon insertion library covering 10% of the genome (Davis *et al*. 2002) using GdA in six environmental conditions (37°C, 41°C, NaCl, tunicamycin, caspofungin and fluconazole).	Total = 226	Hsp90 network is environmentally contingent. GO term enrichment dependent on screen condition. GO term enrichment for macromolecular complexes, protein complexes, protein modification processes, biopolymer modification, post-translational protein modifications and kinases. Degree of connectivity was associated with being up or down-stream of Hsp90. High-connectivity interactors (*CKB1*, *CKB2* and *AHR1*) regulate Hsp90, low-connectivity interactors require Hsp90 for stability and function. *CKB1* and *CKB2* affect phosphorylation of Hsp90 and Cdc37 and protein levels of Hsp90, Cdc37 and Hog1. *AHR1* regulates *HSP90* expression and morphogenesis. ∼17% of network is conserved with *S. cerevisiae*.	Diezmann *et al*. (2012)
*C. albicans*	CGSL screen of two homozygous deletion mutant libraries covering 13% of the genome (Homann *et al*. 2009; Noble *et al*. 2010) using GdA and the same conditions as before (Diezmann *et al*. 2012).	Total = 158	Most Hsp90 interactors detected in caspofungin test condition (86/158). *ERG5* and *STT4* interactors showed increased cellular demand for Hsp90 and mutants had decreased virulence in a macrophage model of infection. *STT4* increases cellular demand for Hsp90 by perturbing actin, whereas deletion of *ERG5* disrupts the ergosterol synthesis pathway and destabilizes the cell wall, increasing cell stress and therefore demand for Hsp90.	O'Meara *et al*. (2016a)
*C. albicans*	Affinity purification (AP-MS) on *HSP90^E36A^*-GFP, *HSP90^E36A^-TAP* and C-terminally TAP-tagged cochaperones (Aha1, Cdc37, Cpr7, Cpr7, Cns1, Hch1, Sba1, Sti1 and Sgt1) grown at 30˚C.The E33A homologous allele E36A stabilizes transient Hsp90 interactions.SILAC to compare Hsp90 competent to Hsp90 inhibited (GdA) or Hsp90-repressed (*tetO-HSP90*) cells.	AP-MS = 188SILAC on GdA treated cells = 505SILAC on tetracycline-repressed cells = 629overlap = 400 proteins found in GdA and tet-repressed cells.	Physical interactors (AP–MS) differed between tagged Hsp90 and co-chaperones expanding the Hsp90 network considerably. GO term enrichment for 20S proteome, kinases, P-bodies, stress granules and RNA binding. The Hsp90 physical interactome is modulated by antifungal drug stress. Depleting several protein interactors that form stress granules or p-bodies caused increased sensitivity to caspofungin. These proteins were dependent on Hsp90 for their stability, aggregation and localization.	O'Meara *et al*. (2019)
*Yarrowia lipolytica*	*Saccharomyces* *cerevisiae* strain *hsp82∆ hsc82∆* heterologously expressing *HSP90* from *Yarrowia lipolytica*, *Naumovozyma castellii*, *Kluyveromyces lactis*.Phenotypic characterization, experimental evolution, sequence analysis of evolved strains.	51 genetic interactions in strain expressing Ylip-HSP90	Orthologous gene replacement reveals functional divergence of Hsp90 among different yeast species. Expressing *Ylip*-*HSP90* causes decreased fitness in many conditions but increased fitness in hyper-saline solutions. *Ylip-HSP90* expressing cells evolved gain-of-function mutations in other genes to compensate for loss of *Scer*-Hsp90 function. *Ylip-HSP90* interactors predominantly genes with Hsp90-related functions.	Koubkova-Yu, Chao and Leu (2018)

### Hsp90 networks in *S. cerevisiae*

Probably the most thorough examination of the fungal Hsp90 network landscape has been achieved in *S. cerevisiae*. Deploying this yeast's extensive toolbox, which includes loss-of-function mutant libraries (Table 1), epitope-tagged libraries (Janke *et al*. 2004; Howson *et al*. 2005) and genome-scale collections suitable for Y2H screens (Uetz *et al*. 2000), provided a comprehensive overview of the eukaryotic Hsp90 interaction landscape. Key findings of the six screens conducted in *S. cerevisiae* are highlighted in Table 2. Based on these screens, several general features of Hsp90 biology can be concluded.

Hsp90 is a network hub that interacts with at least 10% of the proteome (Zhao *et al*. 2005). Genome-scale Hsp90 network data have not just yielded lists of genes and proteins that function in the same pathways and complexes as Hsp90, but further analyses of these extensive lists of Hsp90 clients provided important insights into Hsp90’s role in genome evolution (Zhao *et al*. 2005; Gong *et al*. 2009). Comparative analyses of evolutionary rates of *S. cerevisiae* Hsp90 clients with their homologs in the close relative *S. paradoxus* showed that Hsp90 clients diverged faster due to Hsp90’s ability to buffer destabilizing mutations (Koubkova-Yu, Chao and Leu 2018; Alvarez-Ponce *et al*. 2019). Furthermore, gene/genome duplication is an important pillar of genetic diversification as it produces new genetic material that can take on a new function, share the function of the predecessor, or be lost. The molecular mechanisms involved in these processes are not yet fully understood. The ancestor of *S. cerevisiae* underwent whole genome duplication (Langkjær *et al*. 2003) and *S. cerevisiae* thus provides an ideal testing ground to determine Hsp90’s role in the fate of duplicated genes. Indeed, comparing the evolutionary rates of Hsp90 clients with those of their non-client paralogs revealed that Hsp90 clients evolved faster and Hsp90 thus facilitates the divergence of gene duplicates (Lachowiec *et al*. 2013). Hence, Hsp90 is an important contributor to genome diversification.

Hsp90 interactors are involved in many fundamental cellular processes, therefore, inhibition or depletion of Hsp90 results in a multitude of phenotypes. The most extensive *S. cerevisiae* screen to date, combining Y2H, TAP-MS, SGA and CGSL screens, showed that at 30˚C >10% of interactors function in transcription, cellular fate, protein post-translational modifications, metabolism, cellular transport and the cell cycle and DNA processing (Zhao *et al*. 2005). This finding was supported by a CGSL screen comparing Hsp90 genetic interactors from cells grown at 30 and 37˚C (McClellan *et al*. 2007). Here, Hsp90 interactors are 2-fold enriched for genes involved in nuclear organization, protein binding, signal transduction and pseudohyphal growth. The second screen, furthermore showed that Hsp90 genetic interactors differ with the test environment (McClellan *et al*. 2007) and interactor profiles in cells that were grown in more similar temperatures are more similar to each other (Franzosa *et al*. 2011). Thus, the Hsp90 interaction network is environmentally responsive.

Further, genome-scale *S. cerevisiae* Hsp90 network screens have identified novel Hsp90 co-chaperones, which differ from traditional co-chaperones that are essential for Hsp90 activity, client specificity and directionality of the chaperone cycle (Zuehlke and Johnson 2010). Co-chaperones identified as part of genomic screens for Hsp90 interactors have established links between Hsp90 and specific cellular pathways, such as epigenetic gene regulation (Zhao *et al*. 2005). While rich in discovery, these findings are limited to one species and may not be necessarily transferable to species with other evolutionary trajectories and ecologies.

### Hsp90 networks in *C. albicans* comprise novel regulators of Hsp90 and fungal virulence

To reveal more detail of Hsp90’s role as a regulator of virulence traits in *C. albicans*, the Hsp90 genetic interaction network was mapped using the transposon insertion mutant library (Davis *et al*. 2002). In a CGSL screen, mutants were screened for loss of viability in response to Hsp90 inhibition in six environmental conditions: two different temperatures, mild osmotic stress and three common antifungal drugs (Diezmann *et al*. 2012). Most interactions were detected under just one or two experimental conditions. Only very few interactors were detected during exposure to five or six different conditions. Further analyses showed that high-connectivity interactors, such as Ckb1, are required for Hsp90 phosphorylation and expression, while various low-connectivity interactors were shown to be Hsp90 clients. The experimental set-up of the initial screen was applied to two more *C. albicans* libraries, the Homann TF library and the Noble deletion mutant library (Table 1), whose overlap with the transposon insertion mutant library is limited to 25 mutants (Fig. 3), bringing coverage of the *C. albicans* genome to ∼20%. Here, a similar pattern of high- and low-connectivity interactors was observed (O'Meara *et al*. 2016a). A total of two high-connectivity interactors, *ERG5* and *STT4*, were further characterized. Lack of *ERG5* resulted in hypersensitivity to Hsp90 inhibitors, due to the additional cell stress of a destabilized cell wall and loss of *STT4* increases cellular demand for Hsp90 due to defects in actin organization. Mapping Hsp90 networks in *C. albicans* not only contributed to a better understanding of how the chaperone network modulates *C. albicans* virulence traits (O'Meara, Robbins and Cowen 2017) but revealed another feature of Hsp90 interactors. High-connectivity interactors, those that are detected as essential for growth during most test conditions, affect Hsp90 expression, phosphorylation and function (Diezmann *et al*. 2012; O'Meara *et al*. 2016b). Low-connectivity interactors, those detected at specific environmental conditions, depend on Hsp90 for stability and function (Diezmann *et al*. 2012).

Given the limitations associated with targeting fungal Hsp90, its interactors could provide novel avenues to reducing fungal virulence. To demonstrate just how broadly Hsp90 interactors are involved in different *C. albicans* virulence factors, three were selected for a more detailed review, two of which, *AHR1* and *ERG5*, were identified as high connectivity interactors. The third, *CKA2*, was initially identified as a low-connectivity interactor but was later found to be critical for phosphorylation of a Hsp90 serine residue and modulating Hsp90 is a hallmark of a high-connectivity interactor.

The *Candida*-specific zinc cluster TF Ahr1 (Table 2) activates numerous genes required for fundamental processes of virulence, including adhesion, hyphal growth and biofilm formation (Askew *et al*. 2011) as well as *HSP90* expression (Diezmann *et al*. 2012). Ahr1 furthermore acts as repressor of the white-to-opaque transition (Wang *et al*. 2011) by being one of three core regulators of the white cell regulatory network. This network comprises 179 genes, 93 of which are activated by Ahr1 (Hernday *et al*. 2013). Most recently, it was shown that Ahr1 also activates expression of *ECE1* in hyphae (Ruben *et al*. 2020). *ECE1* is the most abundant transcript in hyphae and the precursor of Candidalysin, the first fungal cytolytic toxin to be identified. Candidalysin is critical for mucosal pathogenesis (Moyes *et al*. 2016). Given how many aspects of *C. albicans* virulence are controlled by Ahr1, it is not surprising that the *ahr1∆/∆* mutant strain displayed attenuated virulence in a murine model of systemic infection (Askew *et al*. 2011).

Erg5 (Table 2) is part of the ergosterol biosynthetic pathway, which is a prominent drug target making Erg5 itself a prominent component of antifungal drug resistance. Deletion of this C-22 sterol desaturase (P450 cytochrome) results in accumulation and integration of different sterol intermediates into the cell membrane, which causes Hsp90 stress (O'Meara *et al*. 2016b). Deletion of *ERG5* also renders *S. cerevisiae* cells resistant to polyene antifungals, such as nystatin (Parks *et al*. 1985). Mutations in *ERG5* and *ERG11*, as identified in a clinical isolate of *C. albicans* from a patient with recurrent oral candidosis, resulted in multi-drug resistance. The strain was reported to be resistant against the most commonly deployed class of antifungals, the azoles and the last-line antimycotic Amphotericin B, severely compromising the antifungal armamentarium available to treat this patient (Martel *et al*. 2010).

The tetrameric kinase Ck2 is not well-characterized in *C. albicans*, but plays a central role in mammalian regulation of cell proliferation and DNA damage repair (Filhol and Cochet 2009). Ck2’s catalytic subunit Cka2 is required for invasion of oral epithelial cells (Chiang *et al*. 2007) and a mutant lacking *CKA2* displays increased resistance to fluconazole (Bruno and Mitchell 2005). The latter phenotype could be explained by the lack of phosphorylation of serine residue 530 in *C. albicans* Hsp90 (Alaalm *et al*. 2021). This phospho-switch regulates Hsp90 stability and the expression of various virulence traits, including drug resistance. Phosphorylation of S530 results in a loss-of-function phenotype, as exemplified by susceptibility to fluconazole, filamentous growth and increased susceptibility to thermal stress. Thus, Cka2 is a repressor of Hsp90 function that requires Hsp90 chaperoning for stability (Diezmann *et al*. 2012).

Further dissecting the functions of Hsp90 interactors identified in networking mapping efforts, will provide new insights into the molecular pathways through which this protein hub governs fungal virulence.

### Core network of Hsp90 interactors comprises key regulators of the environmental stress response

Comparing Hsp90 networks from different species has revealed limited overlap between Hsp90 genetic interactors. Only ∼17% of interactors are conserved between *S. cerevisiae* and *C. albicans* (Zhao *et al*. 2005; Diezmann *et al*. 2012). However, a core of conserved Hsp90 interactors is beginning to emerge. Unsurprisingly, with Hsp90 being a stress-responsive chaperone, core interactors are also involved in stress-response pathways, more specifically several are stress-responsive kinases. In total, two examples of core Hsp90 client kinases are components of the CWIP, which is required for survival during thermal stress. Protein kinase C (Pkc1), which activates the mitogen-activated protein kinase (MAPK) cascade that is integral to the CWIP, is stabilized by Hsp90 in *C. albicans* (Caplan *et al*. 2018), as is the *A. fumigatus* homolog PkcA (Rocha *et al*. 2021). Mkc1, a MAPK of the CWIP, is stabilized by Hsp90 in *C. albicans* (Lafayette *et al*. 2010), in *S. cerevisiae* (Slt2; Millson *et al*. 2005) and *A. fumigatus* (MpkA; Rocha *et al*. 2021). Yet, Hsp90 core interactors are not restricted to CWIP kinases. Hog1, which regulates the osmolarity signaling pathway (Schüller *et al*. 1994) is stabilized by Hsp90 in *S. cerevisiae* (Millson *et al*. 2005) and *C. albicans* (Diezmann *et al*. 2012). Interestingly, even the human homolog, p38, is stabilized and activated by Hsp90 in murine cardiomyocytes (Ota *et al*. 2010) and human sperm (Sun *et al*. 2021). Understanding core Hsp90 interactors may shed new light onto the early days of evolution of this intricate and divergent network.

Beyond experimental mapping and validation of Hsp90 interactions, existing PPI databases, such as the STRING and BioGRID (https://string-db.org/; https://thebiogrid.org/; von Mering *et al*. 2005; Oughtred *et al*. 2021) can be mined for Hsp90 interactions. Querying the STRING database for PPIs between different molecular chaperones in *S. cerevisiae*, *C. albicans*, *A. fumigatus* and *C. neoformans*, revealed that protein interactors have diverged between the different species (Horianopoulos and Kronstad 2021). This is supported by a comparison between experimental data of Hsp90 interactors from two different studies in *S. cerevisiae* (Zhao *et al*. 2005) and *C. albicans* (Diezmann *et al*. 2012), which showed that less than 20% of interactors are shared between these two species. Yet, to fully understand the degree of divergence between Hsp90 chaperone networks in different species, network assays need to be done under comparable conditions.

## CONCLUSIONS AND OUTLOOK

Being able to map Hsp90 interaction networks in diverse fungal species facilitates detection of signatures of evolution. Comparing Hsp90 networks mapped in *S. cerevisiae* that commonly lives on fruit and in the soil, with those gleaned from *C. albicans*, a common human commensal and opportunistic pathogen, will identify common interactors due to shared ancestry and those that evolved in response to selection exerted by the environmental niche. Comparisons between Hsp90 networks in different species will furthermore provide novel insights into general features of network biology, allowing network dynamics and properties, such as degrees of connectivity, to be established more robustly. Also, experimental identification of Hsp90 interactors has the potential to improve existing databases that allow *in silico* investigations of PPI networks, such as STRING and BioGRID databases (https://string-db.org/; https://thebiogrid.org/; von Mering *et al*. 2005; Stark *et al*. 2006; Oughtred *et al*. 2021). While powerful with regards to the fungal kingdom, these databases are often limited to experimentally validated interactions in *S. cerevisiae*, which are then used to predict interactions of orthologs in other species. With the arrival of experimental data in species other than *S. cerevisiae*, predictions can be refined and improved.

Lastly, research on non-model fungal pathogens, which is hampered by low rates of homologous recombination, is currently being revolutionized by CRISPR-Cas technology (Jinek *et al*. 2012). To date, CRISPR-Cas has been adapted to operate in *C. albicans* (Vyas, Barrasa and Fink 2015; Min *et al*. 2016), *C. parapsilosis* (Lombardi *et al*. 2017), *C. glabrata* (Enkler *et al*. 2016), *C. neoformans* (Arras *et al*. 2016) and *A. fumigatus* (van Rhijn *et al*. 2020). Being able to selectively and efficiently manipulate specific loci, will not only enable expansion of existing libraries, but also facilitate creation of libraries in differing genetic backgrounds with varying environmental origins. Being able to mine population-scale mutant collections will further increase resolution of Hsp90 interaction networks.

The model eukaryote *S. cerevisiae* has been instrumental in extracting fundamental knowledge of the nature of Hsp90 networks. Fungal Hsp90 networks can provide critical insights into the evolution of complex chaperone networks and emergence of pathogenesis.
